# Prognostic and immunological value of *ATP6AP1* in breast cancer: implications for SARS-CoV-2

**DOI:** 10.18632/aging.203229

**Published:** 2021-07-06

**Authors:** Jintian Wang, Yunjiang Liu, Shuo Zhang

**Affiliations:** 1 Department of Breast Surgery, The Fourth Hospital of Hebei Medical University, Hebei, Shijiazhuang 050011, China

**Keywords:** *ATP6AP1*, breast cancer, prognosis, immune infiltration, bioinformatics, SARS-CoV-2, COVID-19

## Abstract

Abnormal ATPase H+ Transporting Accessory Protein 1 (*ATP6AP1*) expression may promote carcinogenesis. We investigated the association of *ATP6AP1* with breast cancer (BC) and COVID-19. The Oncomine, Gene Expression Profiling Interactive Analysis, Human Protein Atlas and Kaplan-Meier plotter databases were used to evaluate the expression and prognostic value of *ATP6AP1* in BC. *ATP6AP1* was upregulated in BC tissues, and higher *ATP6AP1* expression was associated with poorer outcomes. Data from the Tumor Immune Estimation Resource, Tumor-Immune System Interaction Database and Kaplan-Meier plotter indicated that *ATP6AP1* expression correlated with immune infiltration, and that its prognostic effects in BC depended on tumor-infiltrating immune cell subtype levels. Multiple databases were used to evaluate the association of *ATP6AP1* with clinicopathological factors, assess the mutation and methylation of *ATP6AP1*, and analyze gene co-expression and enrichment. The *ATP6AP1* promoter was hypomethylated in BC tissues and differentially methylated between different disease stages and subtypes. Data from the Gene Expression Omnibus indicated that *ATP6AP1* levels in certain cell types were reduced after SARS-CoV-2 infections. Ultimately, higher *ATP6AP1* expression was associated with a poorer prognosis and with higher or lower infiltration of particular immune cells in BC. BC patients may be particularly susceptible to SARS-CoV-2 infections, which may alter their prognoses.

## INTRODUCTION

In women, breast cancer (BC) is one of the leading causes of death, and is the leading cause of cancer-related death [1]. Although early detection and advanced treatment methods for BC are rapidly being developed, further research is needed to clarify the underlying pathways and prognostic factors of BC. Cancer immune surveillance is a critical process whereby the immune system combats tumors [2]; thus, it is especially important to determine the immune escape mechanisms of BC and identify more effective immunotherapeutic targets so that BC patients can be treated more precisely.

The tumor microenvironment contributes significantly to tumor development, and is characterized by an acidic pH. ATPase H+ Transporting Accessory Protein 1 (ATP6AP1) is a component of a multi-subunit enzyme within Vacuole ATPase (V-ATPase) [3], and deficiencies in this protein can cause immunodeficiency, hepatopathy, cognitive impairment and abnormal protein glycosylation [4]. Due to its function as a proton pump, V-ATPase can help cancer cells excrete excess H+, reverse the transmembrane proton gradient and form a highly acidic extracellular environment while avoiding apoptosis [5]. A recent study indicated that salivary autoantibodies against ATP6AP1 could be used as biomarkers for the early detection of BC [3]. Therefore, ATP6AP1 may alter the immune microenvironment of BC and the prognoses of BC patients.

Severe acute respiratory syndrome coronavirus 2 (SARS-CoV-2), a novel coronavirus of the family Coronaviridae, was identified after the outbreak of the COVID-19 pandemic [6]. Other coronaviruses such as Middle East Respiratory Syndrome-related coronavirus in 2012 and Severe Acute Respiratory Syndrome coronavirus (SARS, also known as SARS-CoV-1) in 2002 have led to massive epidemics across certain continents, and have especially impacted cancer patients [7, 8]. Given the increased incidence rate of SARS-CoV-2, it is likely to co-exist with humans for a long time, like influenza. A previous report indicated that ATP6AP1 can function as a bait for the SARS-CoV-2 nsp6 non-structural protein [9]. The interactions of cancer-related proteins with viruses may alter the prognoses of cancer patients [10]; however, the effects of ATP6AP1 on the prognoses of BC patients during the COVID-19 pandemic have not been described.

In this study, we used public datasets to evaluate *ATP6AP1* levels in BC tissues and to determine their correlation with clinicopathological features and patient prognoses. We also investigated tumor-immune infiltration and its association with the prognostic value of *ATP6AP1* in BC. Additionally, we determined the genes and pathways associated with *ATP6AP1* to clarify its function in BC.

## RESULTS

### *ATP6AP1* levels in BC patients

**Figure 1:**
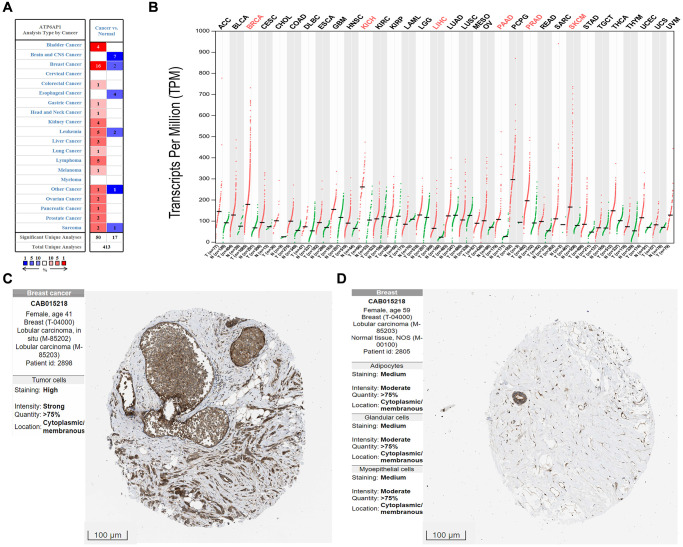
***ATP6AP1* levels in cancer tissues compared with normal tissues.** (**A**) *ATP6AP1* mRNA levels in samples from the Oncomine database. The numbers in the colored squares represent the number of involved studies. The different colors correspond to different *ATP6AP1* levels, with red representing high expression and blue representing low expression. The darker the red color, the higher the expression, and the darker the blue color, the lower the expression. (**B**) *ATP6AP1* mRNA levels in samples from the GEPIA database. Red indicates significant results. (**C**, **D**) ATP6AP1 protein levels in normal and cancerous breast tissues based on immunohistochemistry data from the HPA database (antibody: CAB015218, provided by Origene. Dilution: 1:30).

We first compared *ATP6AP1* expression between BC tissues and normal tissues using the Oncomine database (Figure 1A). *ATP6AP1* mRNA levels were significantly greater in BC tissues than in normal tissues in multiple datasets (*P* < 0.05). Then, we used Gene Expression Profiling Interactive Analysis (GEPIA) to compare *ATP6AP1* mRNA levels between BC and normal tissues based on RNA sequencing data from The Cancer Genome Atlas (TCGA) and the Genotype-Tissue Expression (GTEx) database (Figure 1B). *ATP6AP1* mRNA levels were also significantly upregulated in BC tissues in this analysis (all *P* < 0.05). We then assessed ATP6AP1 protein levels using the Human Protein Atlas (HPA) database, and found that ATP6AP1 was moderately expressed in normal breast tissues, but moderately or highly expressed in BC tissues. The representative immunohistochemistry results from the HPA database in Figure 1C and 1D illustrate that ATP6AP1 protein levels were greater in BC tissues than in normal tissues. Others were showed in the Supplementary Figure 1.

### Prognostic potency of *ATP6AP1* in BC

**Figure 2:**
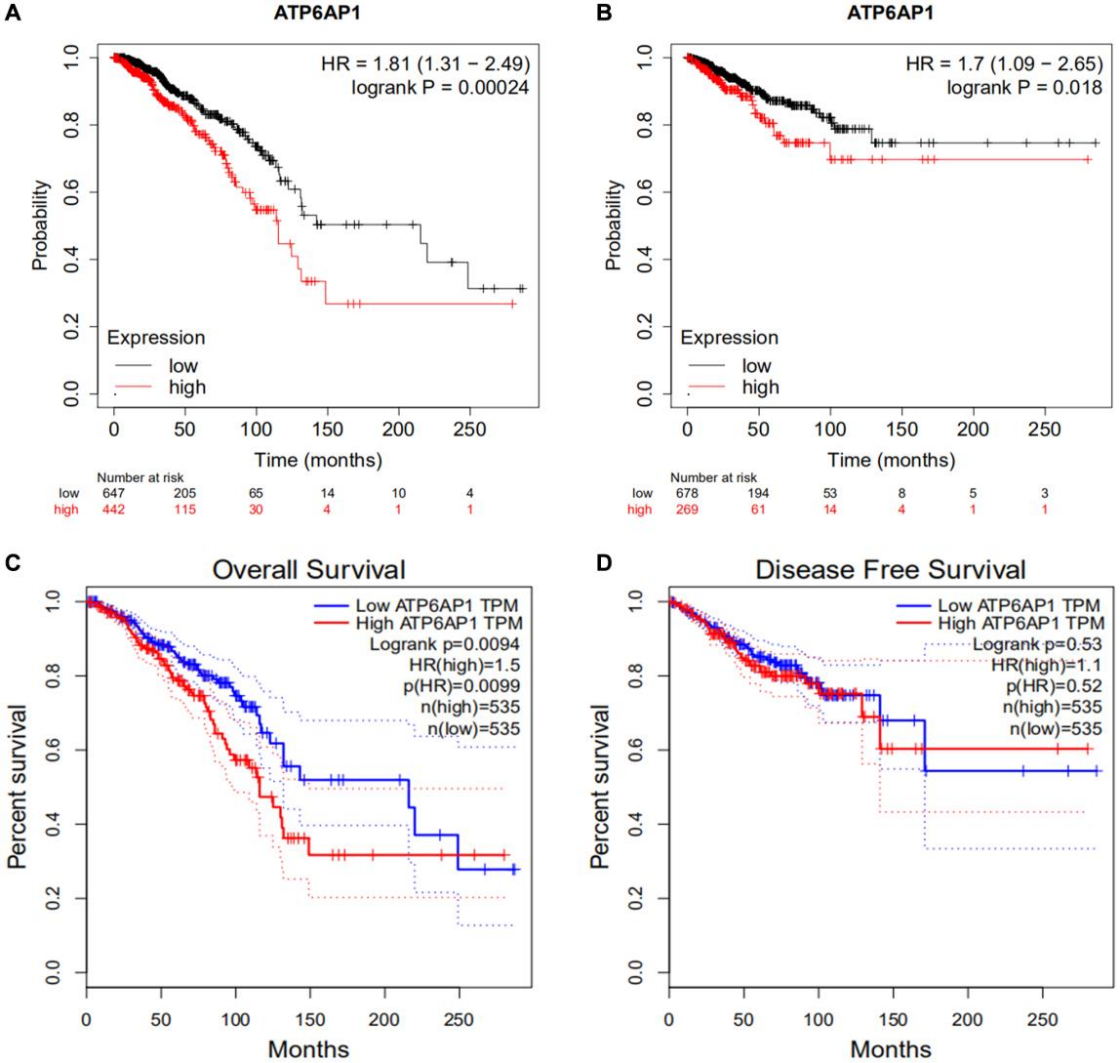
**Survival curves in BC patients with higher and lower *ATP6AP1* levels.** (**A**, **B**) OS and RFS of BC patients from the Kaplan-Meier plotter database (*n* = 1089 and *n* = 947, respectively). (**C**, **D**) OS and DFS curves of BC patients from GEPIA (*n* = 1070 and *n* = 1070, respectively).

To determine the relationship between *ATP6AP1* expression and BC prognosis, we used the Kaplan-Meier Plotter to determine the overall survival (OS) and relapse-free survival (RFS) or disease-free survival (DFS) of BC patients who were separated into two groups (high and low) based on the median *ATP6AP1* level. As shown in Figure 2A and 2B, higher *ATP6AP1* expression was associated with a poorer BC prognosis (OS hazard ratio = 1.81, log-rank *P* = 0.00024; RFS hazard ratio = 1.7, log-rank *P* = 0.018). Data from TCGA in GEPIA confirmed that higher *ATP6AP1* expression was associated with a significantly poorer prognosis in BC patients (all *P* < 0.05; Figure 2C and 2D).

### Correlation of *ATP6AP1* levels with tumor-infiltrating immune cell (TIIC) and immune cell marker levels in BC

**Figure 3:**
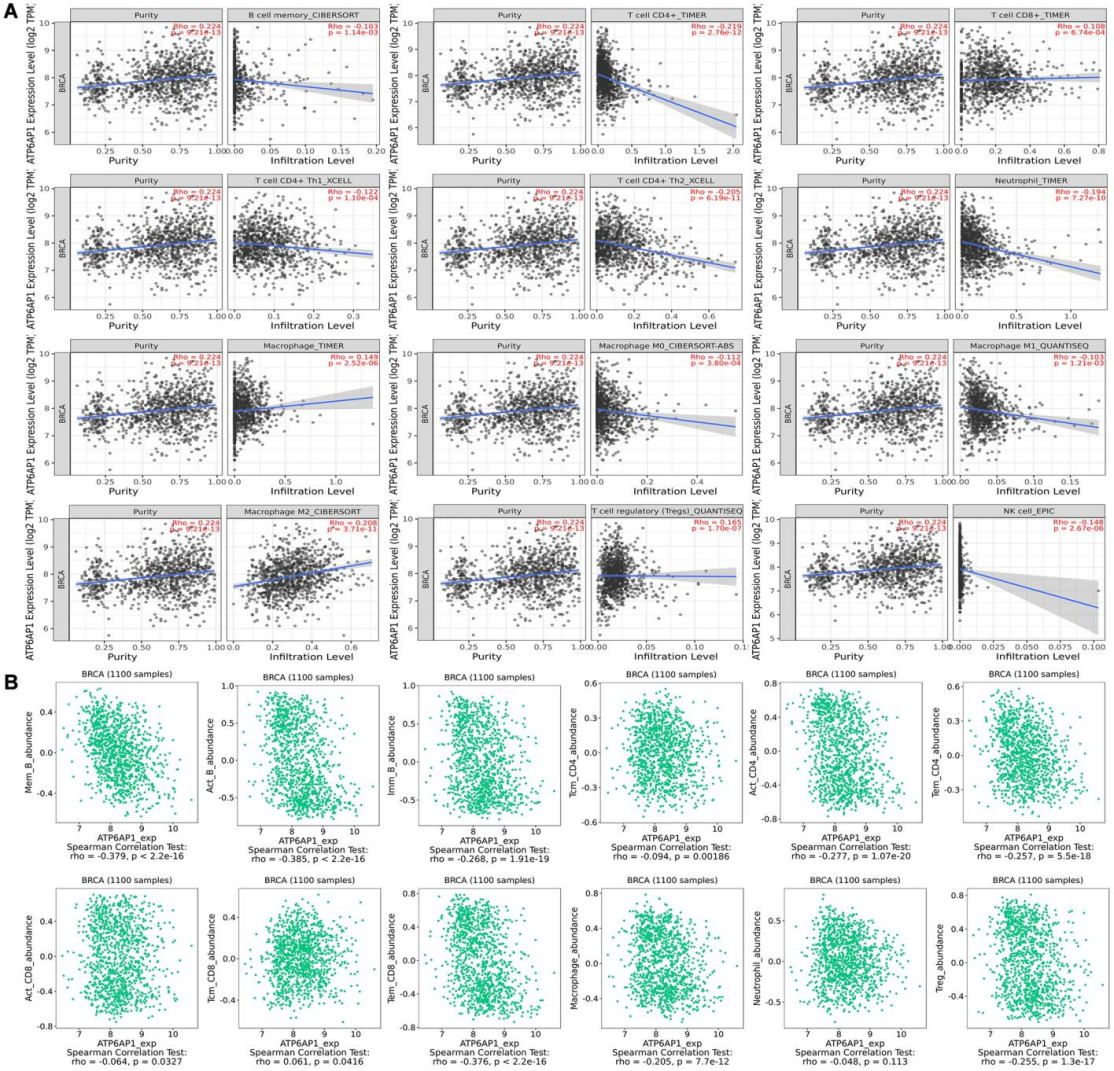
**Correlation of *ATP6AP1* expression with immune infiltration in BC samples from TIMER and TISIDB.** The correlation of *ATP6AP1* levels with the infiltrating levels of B cells, CD4+ T cells, CD8+ T cells, macrophages, Tregs, natural killer (NK) cells and neutrophils in BC samples from TIMER (**A**) and TISIDB (**B**), respectively (*n* = 1100). The CIBERSORT, TIMER, XCELL, QUANTISEQ and EPIC in Figure represents the algorithm which the database used.

We then investigated whether *ATP6AP1* expression correlated with the levels of TIICs and immune cell markers in BC through Tumor Immune Estimation Resource (TIMER). Correlation analyses revealed that *ATP6AP1* levels correlated remarkably with tumor purity and to varying degrees with immune cell levels. *ATP6AP1* levels were negatively associated with the levels of CD4+ T cells (Rho = –0.219, *P* = 2.76e-12), neutrophils (Rho = –0.194, *P* = 7.27e-10), type 2 T helper (Th2) cells (Rho =-0.205, *P* = 6.19e-11) and natural killer cells (Rho = –0.148, *P* = 2.67e-06) in BC tissues (Figure 3A). However, *ATP6AP1* levels were positively associated with the levels of Tregs (Rho = 0.165, *P* = 1.07e-07), macrophages (Rho = 0.149, *P* = 2.52e-06) and M2 macrophages (Rho = 0.208, *P* = 3.71e-11). The levels of memory B cells, CD8+ T cells, type 1 T helper (Th1) cells and M0/M1 macrophages were weakly associated with *ATP6AP1* levels.

Next, we used the Tumor-Immune System Interaction Database (TISIDB) to further assess the relationship between *ATP6AP1* levels and TIIC levels in BC (Figure 3B). Notably, *ATP6AP1* levels correlated negatively with B cell, CD4+ T cell, Treg and macrophage levels, and exhibited a weak negative correlation with CD8+ T cell levels (all *P* < 0.05). However, *ATP6AP1* levels did not correlate significantly with neutrophil levels (*P* = 0.113). Combining the results from Figure 3A and 3B, CD4+ T cells, Tregs and macrophages may strongly influence *ATP6AP1* expression in BC, thus altering the prognoses of BC patients.

We subsequently determined the correlations between the levels of diverse immune cell markers and *ATP6AP1*, while removing the influence of tumor purity. *ATP6AP1* levels exhibited significant negative correlations with B cell, CD8+ T cell, macrophage and Treg surface marker levels (all *P* < 0.05; Table 1). Interestingly, no significant correlations between the levels of these immune cell markers and *ATP6AP1* remained when we removed the influence of the patient’s age. Nevertheless, *ATP6AP1* expression may generally be associated with immune infiltration in BC.

**Table 1:** Correlation analysis between *ATP6AP1* and immune cell type markers in TIMER database.

**Cell type**	**Gene markers**	**None**		**Purity**		**Age**	
		**COR**	***P***	**COR**	***P***	**COR**	***P***
B Cells	CD19	–0.224	5.12E-14	–0.121	1.31E-04	0.067	5.57E-01
	FCRL2	–0.224	5.75E-14	–0.128	5.48E-05	–0.066	5.65E-01
	CD38	–0.214	7.37E-13	–0.116	2.52E-04	0.025	8.27E-01
CD8+T Cells	CD27	–0.222	1.08E-13	–0.104	1.02E-03	–0.072	5.30E-01
	CD3D	–0.245	1.85E-16	–0.136	1.81E-05	–0.097	3.98E-01
	CD8A	–0.169	1.61E-08	–0.059	6.48E-02	–0.138	2.30E-01
Neutrophils	CXCR2	–0.027	3.65E-01	0.043	1.79E-01	–0.060	5.99E-01
	SELL	–0.094	1.84E-03	0.032	3.10E-01	–0.001	9.94E-01
	FCGR3B	0.033	2.67E-01	0.069	2.98E-02	0.083	4.70E-01
Macrophages	CD14	–0.178	2.86E-09	–0.110	5.08E-04	–0.216	5.77E-02
	CD163	–0.078	9.43E-03	0.007	8.19E-01	–0.014	9.05E-01
	CD84	–0.013	6.58E-01	0.086	6.84E-03	–0.011	9.25E-01
Dendritics	CCL18	–0.208	3.56E-12	–0.156	8.35E-07	–0.076	5.07E-01
	CD209	–0.128	1.97E-05	–0.030	3.38E-01	–0.121	2.93E-01
	LYZ	–0.149	6.65E-07	–0.043	1.80E-01	–0.160	1.61E-01
NK cells	CD69	–0.163	5.54E-08	–0.036	2.59E-01	–0.168	1.41E-01
	NKG7	–0.231	7.68E-15	–0.124	9.17E-05	–0.182	1.11E-01
Th1 cells	CCR1	–0.111	2.37E-04	–0.035	2.66E-01	–0.132	2.48E-01
	CCR5	–0.164	4.44E-08	–0.050	1.17E-01	–0.165	1.49E-01
	CXCR3	–0.189	2.93E-10	–0.074	2.01E-02	–0.102	3.73E-01
Treg	BIRC3	–0.249	5.43E-17	–0.152	1.49E-06	0.073	5.27E-01
	CCR4	–0.070	2.09E-02	0.060	5.82E-02	0.082	4.76E-01
	FOXP3	–0.158	1.32E-07	–0.062	4.95E-02	0.081	4.79E-01
Monocytes	CD86	–0.133	9.24E-06	–0.035	2.69E-01	–0.177	1.21E-01
	MNDA	–0.087	3.83E-03	0.028	3.86E-01	–0.127	2.69E-01

^a^COR, rho value of Spearman’s correlation; Purity, correlation adjusted by purity; ^b^Age, correlation adjusted by age.

### Survival analysis based on *ATP6AP1* expression in BC patients with different immune cell subtype levels

**Figure 4:**
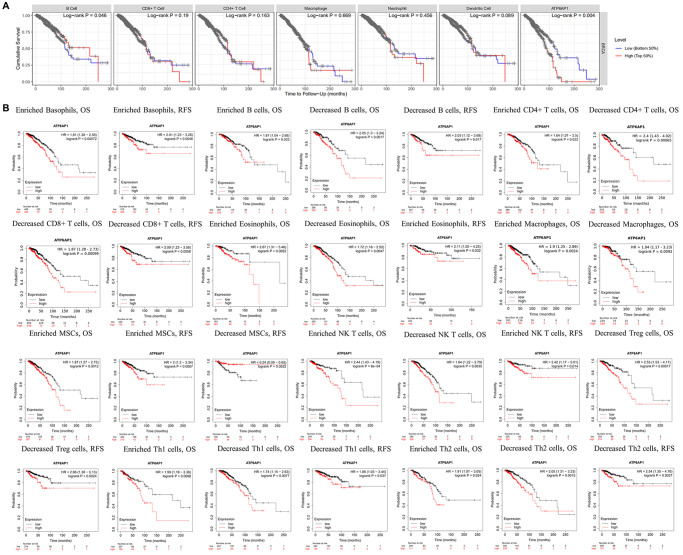
**Survival curves based on *ATP6AP1* levels stratified according to TIIC levels in BC samples from TIMER and Kaplan-Meier Plotter.** (**A**) Data from TIMER (*n* = 1100). (**B**) Data from Kaplan-Meier Plotter.

Since higher *ATP6AP1* expression was associated with a poorer prognosis in BC patients, we used the TIMER to determine whether this association depended on the abundance of immune infiltrates in BC tissues (Figure 4A). *ATP6AP1* expression-based clinical outcomes correlated significantly with B cell levels (log-rank *P* = 0.046). We then used Kaplan-Meier Plotter to assess the relationship between *ATP6AP1* levels and clinical outcomes after stratifying patients according to the levels of other types of TIICs (Figure 4B). BC patients with higher *ATP6AP1* levels had poorer prognoses, whether they had enriched or reduced levels of B cells, CD4+ T cells, eosinophils, macrophages, mesenchymal stem cells, natural killer T cells, Th1 cells or Th2 cells (all *P* < 0.05). On the other hand, BC patients with higher *ATP6AP1* levels had poorer prognoses if they had enriched basophil levels, reduced CD8+ T cell levels or reduced Treg levels (all *P* < 0.05), but not if they had reduced basophil levels, enriched CD8+ T cell levels or enriched Treg levels. Thus, immune infiltration may partly explain the poorer prognoses of BC patients with higher *ATP6AP1* levels.

### The clinicopathological significance of *ATP6AP1* expression and methylation in BC

**Figure 5:**
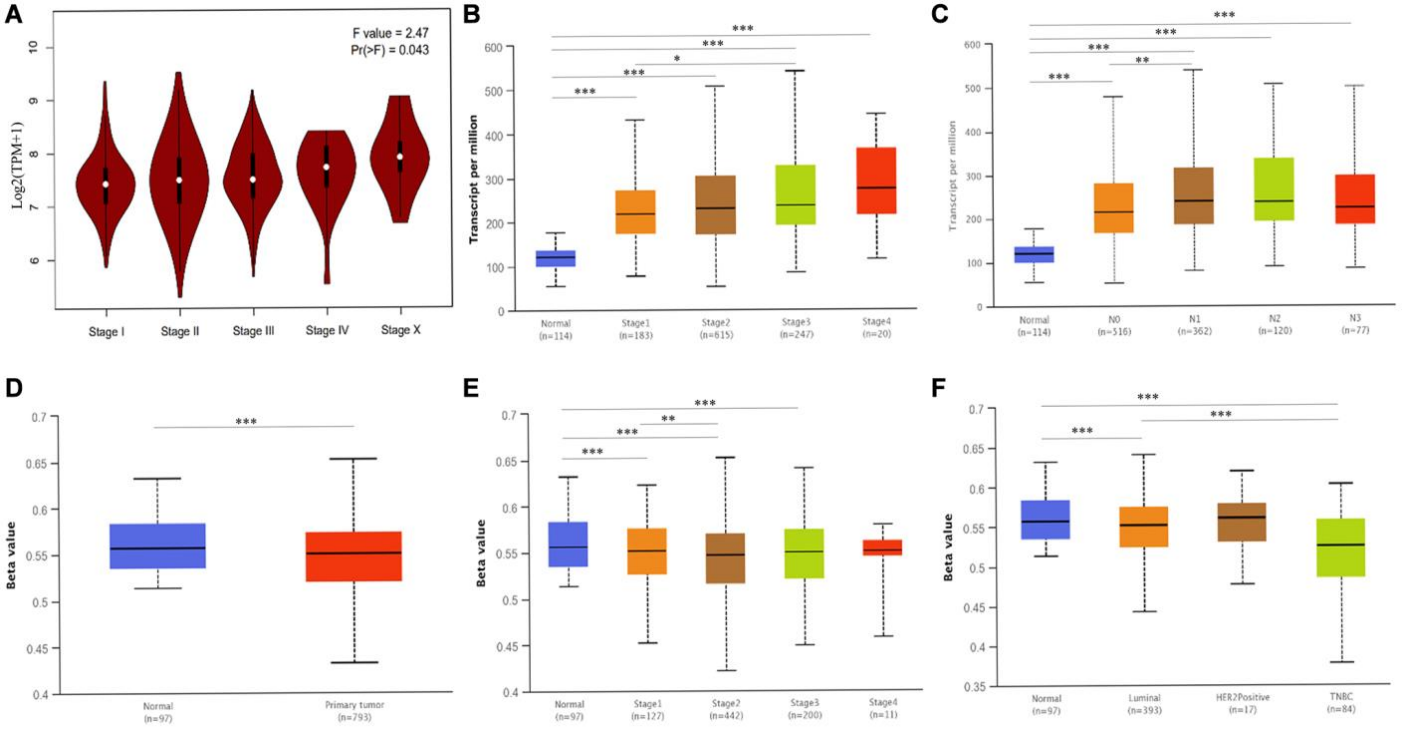
**Associated clinicopathological factors and promoter methylation levels of *ATP6AP1* in BC.** (**A**) *ATP6AP1* levels in various pathological substages of BC in GEPIA. (**B**) *ATP6AP1* levels in different disease stages of BC in UALCAN. (**C**) The correlation between lymph node metastasis and *ATP6AP1* expression in BC. (**D**) *ATP6AP1* promoter methylation profiles of different sample types. (**E**) *ATP6AP1* promoter methylation levels of different BC disease stages. (**F**) *ATP6AP1* promoter methylation levels of different major subclasses of BC. ^*^P < 0.05, ^**^P < 0.01, ^***^P < 0.001.

Next, we investigated whether *ATP6AP1* expression was associated with clinical factors in BC patients. *ATP6AP1* levels correlated positively with the pathological stage (Pr(>F) = 0.043) and clinical stage, with an especially pronounced difference between stages 1 and 3 (*P* < 0.05; Figure 5A and 5B). As for lymph node metastasis, *ATP6AP1* levels were significantly greater in N1 than in N0 BC patients (*P* < 0.05; Figure 5C). However, *ATP6AP1* expression had little association with other clinical and pathological factors, as shown in Supplementary Figure 2.

Epigenetic changes in DNA methylation (whether they occur within a single gene or across the genome) are important contributors to tumor initiation and development [11]. Thus, we used the UALCAN database to compare the methylation of the *ATP6AP1* promoter between BC tissues and normal tissues. The *ATP6AP1* promoter was significantly hypomethylated in BC tissues compared with normal tissues (*P* < 0.05; Figure 5D). Moreover, the methylation of the *ATP6AP1* promoter was significantly associated with the subtypes and disease stages of BC, especially differing between luminal and triple-negative BC and between stages 1 and 2 (all *P* < 0.05; Figure 5E and 5F). Thus, hypomethylation of *ATP6AP1* may promote the occurrence and development of BC. We also assessed the genetic variation of *ATP6AP1* in BC tissues, and found that there was approximately 1.4% variation due to fusions, amplifications, deep deletions, missense mutations and truncating mutations of unknown significance (Supplementary Figure 3). These findings suggested that variations in *ATP6AP1* may contribute to BC tumorigenesis.

### *ATP6AP1*-related network and functional analysis in BC

**Figure 6:**
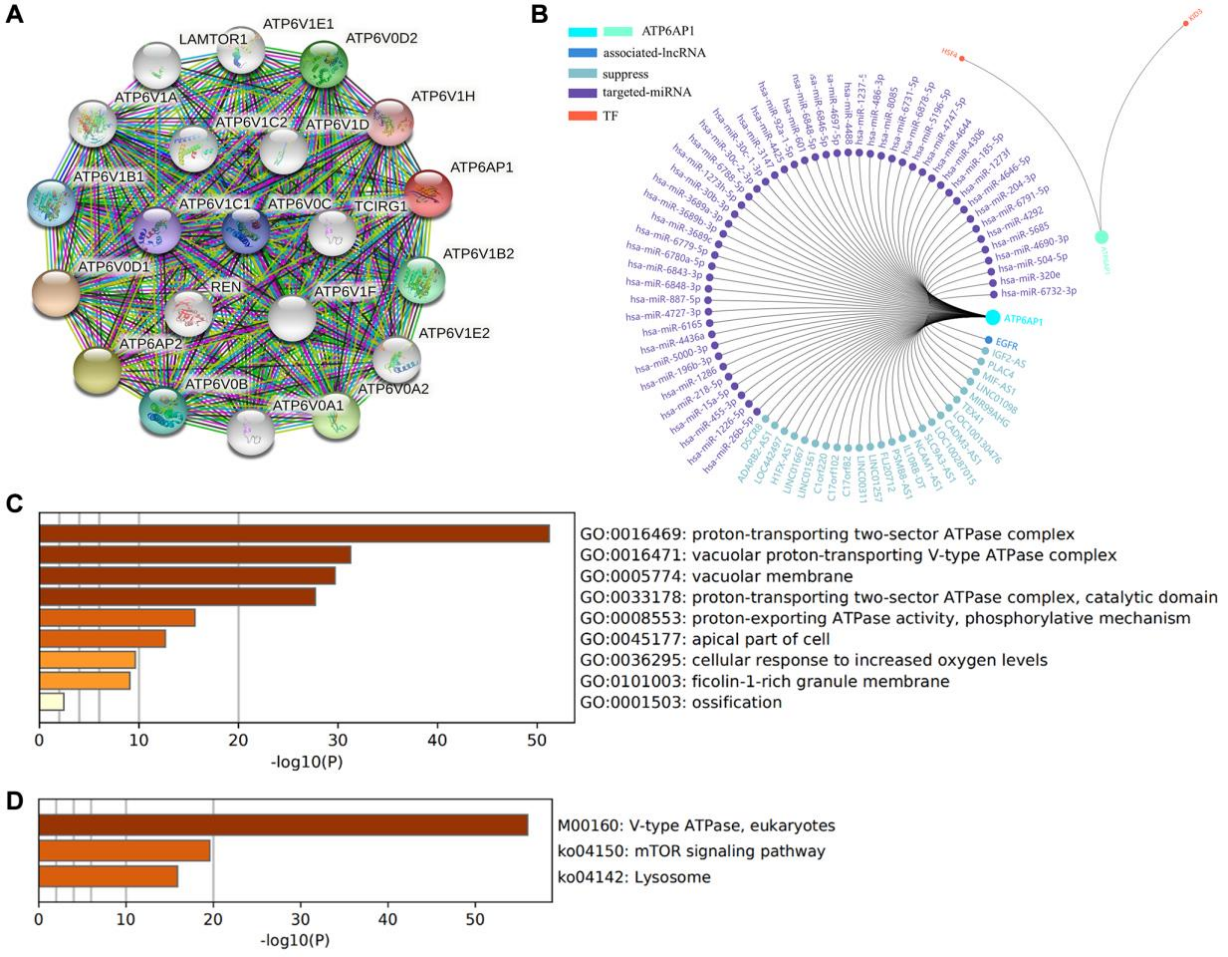
**Functional enrichment and regulatory network analyses of ATP6AP1.** (**A**) The protein-protein interaction network of ATP6AP1. (**B**) Regulatory network analysis conducted in GCBI. (**C**) GO functional analysis. (**D**) KEGG pathway analysis.

To determine the biological significance of *ATP6AP1* in BC, we used the function module of UALCAN to examine genes that were co-expressed with *ATP6AP1* in the BC cohort. The top 48 genes with significant positive or negative correlations with *ATP6AP1* levels are presented in Table 2. We also analyzed the co-expression of proteins with ATP6AP1 in BC using the Search Tool for the Retrieval of Interacting Genes (STRING) database. Twenty-one proteins were significantly co-expressed with ATP6AP1, and these proteins were used to construct a protein-protein interaction network, which contained 21 nodes and 210 edges (Figure 6A). Most of these proteins were components of V-ATPase, except for ATP6AP2, LAMTOR1 (Late Endosomal/Lysosomal Adaptor, MAPK and mTOR Activator 1) and Renin, and all of them have previously been implicated in BC [12, 13].

**Table 2:** The co-expression genes of *ATP6AP1* analyzed by UALCAN.

**Positively Genes**	**Pearson CC**	**Negatively Genes**	**Pearson CC**
*GDI1*	0.71	*RPS27A*	–0.44
*BCAP31*	0.6	*RPS7*	–0.40
*ARF3*	0.57	*RPS19*	–0.39
*P4HTM*	0.56	*CSDA*	–0.38
*TMBIM6*	0.56	*RPL37*	–0.38
*NECAB3*	0.52	*RPS25*	–0.38
*POMGNT1*	0.52	*C11orf75*	–0.37
*TMED4*	0.52	*RPS18*	–0.36
*CETN2*	0.51	*RPL35A*	–0.35
*PIGT*	0.50	*RPL24*	–0.35
*FAM134A*	0.50	*RPS10*	–0.35
*CXorf40B*	0.50	*EEF1B2*	–0.35
*SPRYD3*	0.50	*RPL5*	–0.35
*RELL1*	0.50	*RPL11*	–0.35
*ATP6V0A1*	0.50	*TUBB6*	–0.34
*CXXC5*	0.49	*RPL18A*	–0.34
*RAB5B*	0.49	*RPS6*	–0.34
*HAGH*	0.49	*RPL27A*	–0.34
*PLXNA3*	0.49	*OBFC2A*	–0.33
*RAB3A*	0.49	*RPS12*	–0.33
*ATP6V0B*	0.48	*C6orf145*	–0.33
*LRBA*	0.48	*RPL31*	–0.33
*WFS1*	0.48	*EEF1G*	–0.33
*CDKN2AIPNL*	0.48	*UBE2E3*	–0.33

^a^Pearson CC value: 0·00–0·19 “very weak,” 0·20–0·39 “weak,” 0·40–0·59 “moderate,” 0·60–0·79 “strong,” 0·80–1·0 “very strong.

Next, we evaluated the enriched Gene Ontology (GO) biological functions and Kyoto Encyclopedia of Genes and Genomes (KEGG) pathways of the proteins in this interaction network (Figure 6C and 6D). In terms of biological function, the proteins were mainly associated with the ‘proton-transporting two-sector ATPase complex’, ‘vacuolar proton-transporting V-type ATPase complex’, ‘vacuolar membrane’, ‘proton-transporting two-sector ATPase complex (catalytic domain)’, ‘proton-exporting ATPase activity (phosphorylative mechanism)’, ‘apical part of cell’, ‘cellular response to increased oxygen levels’ and ‘filcolin-1-rich granule membrane’. In the KEGG pathway enrichment results, three pathways were the most likely to represent the function of ATP6AP1 in BC: ‘V-type ATPase (eukaryotes)’, ‘mTOR signaling pathway’ and ‘lysosome’.

We also used the Gene-Cloud of Biotechnology Information (GCBI) database to further explore the regulators of *ATP6AP1* in BC. The long noncoding RNAs (lncRNAs), microRNAs (miRNAs) and transcription factors (TFs) associated with *ATP6AP1* are shown in Figure 6B.

### SARS-CoV-2 infections may reduce the expression of *ATP6AP1*

**Figure 7:**
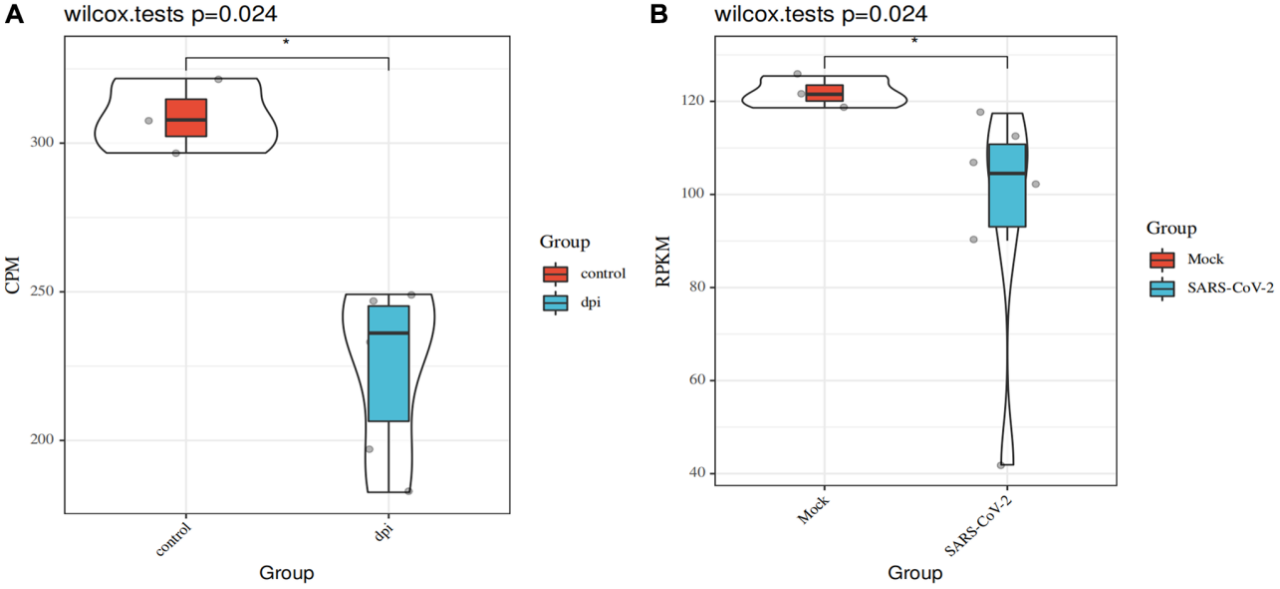
**Changes in *ATP6AP1* levels after SARS-CoV-2 infections.** (**A**) Comparison of *ATP6AP1* levels between the mock (*n* = 3) and SARS-CoV-2-infected groups (*n* = 6) of iAT2 cells from the GSE153277 dataset. Triplicate results are shown for iAT2 cells cultured at the air-liquid interface with a mock virus (mock, *n* = 3), with SARS-CoV-2 one day post-infection (1 dpi, *n* = 3), and with SARS-CoV-2 four days post-infection (4 dpi, *n* = 3). (**B**) *ATP6AP1* levels in the SARS-CoV-2-infected group (*n* = 6) and the mock group (*n* = 3) of hPSC-LOs from the GSE155241 dataset. CPM: counts per million. RPKM: reads per kilobase million.

ATP6AP1 can be used as a bait through which SARS-CoV-2 nsp6 infects people [9]. Thus, we used the GSE153277 and GSE155241 datasets to assess changes in *ATP6AP1* expression after SARS-CoV-2 infections. The results from GSE153277 indicated that *ATP6AP1* levels in induced alveolar type II epithelial-like (iAT2) cells were significantly lower in the SARS-CoV-2-infected group than in the control group (Figure 7A). Likewise, in GSE155241, *ATP6AP1* levels in human pluripotent stem cell-lung organoids (hPSC-LOs) declined significantly after the cells were infected with SARS-CoV-2 (Figure 7B). These findings suggested that *ATP6AP1* levels decrease after SARS-CoV-2 infections.

Tumor tissues expressing high levels of *ATP6AP1* may be particularly susceptible to viral interference. Since SARS-CoV-2 infections can reduce *ATP6AP1* levels, and *ATP6AP1* expression is associated with immune infiltration in BC, SARS-CoV-2 may influence the occurrence, development and prognosis of BC.

## DISCUSSION

In this study, we comprehensively analyzed the involvement of *ATP6AP1* in BC. In the context of the novel coronavirus, it is particularly important to determine the prognoses of cancer patients, especially BC patients. As ATP6AP1 is known to bind to SARS-CoV-2, understanding the contribution of *ATP6AP1* to the development and prognosis of BC is greatly significant. We found that *ATP6AP1* levels were significantly greater in BC tissues than in normal breast tissues, and correlated significantly with the cancer stage and lymph node metastasis status (N1 vs. N0). These results indicated that *ATP6AP1* may be an ideal biomarker for early BC diagnosis and nodal metastasis detection. Moreover, higher *ATP6AP1* expression was associated with poorer OS and DFS in BC patients. As far as we know, this is the first study to report the association of greater *ATP6AP1* mRNA levels with a poorer prognosis in BC patients.

We also found that *ATP6AP1* levels correlated with the levels of TIICs in BC, especially those of CD4+ T cells, Tregs and macrophages. However, in terms of whether the correlations were positive or negative, the results from TIMER and TISIDB differed from one another for Tregs and macrophages. The levels of most immune cell types correlate negatively with tumor purity, so correcting for purity can help to clarify the association of gene expression with immune cell infiltration. Since TIMER corrects for tumor purity, its results are likely to be more accurate, especially given that this database employs six algorithms [14]. Moreover, TISIDB does not contain a subtype analysis for macrophages.

Our results indicated that *ATP6AP1* expression correlated negatively with CD8+ T cell and B cell infiltration, but positively with Treg and macrophage infiltration. Tumor-associated macrophages can dramatically influence tumor initiation and progression [15], and have been associated with a poor prognosis in BC [16]. M2 (anti-inflammatory) tumor-associated macrophages have been implicated in the progression of BC to invasive carcinoma. Regarding T cells, CD8+ T cell infiltration is known to induce an anti-tumor cytotoxic response [17], whereas the prognostic value of CD4+ immune cell infiltration is somewhat controversial. CD4+ T cells comprise at least four lineages – Th1, Th2, Th17 and Treg cells – which differ in their functions. CD4+ Th1 cells have been shown to prevent tumor growth [16], while CD4+ Th2 and CD4+ forkhead box P3+ lymphocytes (Tregs) are considered to promote tumor growth [15, 16, 18]. CD4+ Tregs can suppress host-derived adaptive anti-tumor immunity by inhibiting tumor-specific cytotoxicity [19]. A comprehensive retrospective analysis indicated that a greater density of infiltrating forkhead box P3+ immune cells was associated with poorer OS in BC patients [20]. As for B cells, greater CD20+ B cell infiltration has been associated with a better survival rate in BC patients [21]. Since *ATP6AP1* levels correlated negatively with CD8+ T cell and B cell levels in the present study, high *ATP6AP1* expression in BC tissues may inhibit the cytotoxic response to tumor cells, resulting in poor outcomes. The positive correlation of *ATP6AP1* levels with CD4+ Treg and macrophage levels further explains why patients with higher *ATP6AP1* levels had poorer prognoses, since these TIICs can promote tumor growth. Our findings indicate that *ATP6AP1* may be a key contributor to immune suppression and immune escape, and may worsen the prognoses of BC patients by regulating immune infiltration.

Previous studies have demonstrated that the loss of ATP6AP1 can be carcinogenic; for instance, an inactivating mutation in *ATP6AP1* was proposed to be the driving factor for granular cell tumor development [22]. On the other hand, the overexpression of oncogenes due to hypomethylation is considered to be an important mechanism of carcinogenesis [23], and DNA methylation is a candidate early biomarker of BC progression [24]. The luminal B subtype of BC is characterized by a hypermethylated phenotype, while the basal-like subtype is characterized by hypomethylation [25]. We found that the *ATP6AP1* promoter was markedly hypomethylated in BC tissues compared with normal tissues, and that its methylation level differed significantly between the luminal and triple-negative subtypes. We also observed that *ATP6AP1* promoter methylation differed significantly among BC stages, most significantly between stage 1 and 2. Epigenetic modifications of genes may alter the tumor immune microenvironment and induce strong anti-tumor immune responses [11, 24, 26]. Thus, the hypomethylation of *ATP6AP1* may explain its increased mRNA levels in BC tissues.

Our analysis indicated that *ATP6AP1* expression in certain cell types may be reduced after SARS-CoV-2 infections; thus, its expression in tumor tissues may also be reduced following such infections. Considering that *ATP6AP1* was upregulated in BC tissues, these tissues may be more susceptible to viral interference from SARS-CoV-2. Moreover, in our protein-protein interaction network analysis, one of the proteins found to interact with ATP6AP1 was Renin, the rate-limiting enzyme of the renin-angiotensin system. Renin not only has vital functions in cardiovascular and kidney disease, but also is associated with diverse cancers, especially BC [27]. Angiotensin converting enzyme 2, a key regulator of the renin-angiotensin system, has been identified as a functional receptor for SARS-CoV-2 [28]. Given these findings, it is possible that SARS-CoV-2 could alter *ATP6AP1* expression in BC tissues, thus disturbing the tumor microenvironment and influencing the development and prognosis of BC.

Our GO and KEGG enrichment analyses indicated that the proteins co-expressed with ATP6AP1 were mainly components of V-ATPase and participants in the mammalian target of rapamycin (mTOR) pathway. V-ATPase regulates the extracellular environment of tumor cells and the pH of many intracellular compartments, thus enabling tumor cells to maintain a high metabolic rate and contributing to their autophagy, invasion, migration and drug resistance [5, 29]. Enhanced phosphoinositide 3-kinase/AKT/mTOR signaling has been associated with BC and found to promote drug resistance [30]. Everolimus, which inhibits mTORC1 and induces AKT phosphorylation, has been approved for the treatment of postmenopausal women with hormone receptor-positive and human epidermal growth factor receptor 2-negative advanced BC [31]. Therefore, it is extremely likely that these co-expressed genes and interacting proteins contribute to the tumorigenic effects of *ATP6AP1* in BC.

We also identified the lncRNAs, miRNAs, TFs and downstream genes associated with *ATP6AP1* in BC. A number of these genes are known to be involved in cancer, including the downstream gene epidermal growth factor receptor (*EGFR*), the lncRNAs *DSCR8* and *ADARB2-AS1*, and the TF heat shock factor 4 (*HSF4*). V-ATPase and EGFR can antagonize one another [32]. The induction of *EGFR* under hypoxic conditions has been found to promote cell proliferation and migration, and patients with hypoxic breast tumors and *EGFR* hypomethylation may benefit from *EGFR* inhibition [33]. Unfortunately, the specific mode of interaction between *EGFR* and *ATP6AP1* in BC is still unknown. Dysregulation of the lncRNA *DSCR8* has been observed in uterine cancer, melanoma and liver cancer [34], and upregulation of *ADARB2-AS1* has been detected in human epidermal growth factor receptor 2-positive BC [35]. Knockout or overexpression of either *HSF4* and *HSF2* have been shown to increase the hypoxia-inducible factor-1α expression in MCF-7 BC cells [36], though it is unclear whether *HSF4* directly contributes to the development of BC. These genes are probably part of the regulatory network of *ATP6AP1* in BC; however, their exact functions remain to be determined.

Our study had several limitations. Firstly, the number of normal breast tissue specimens available in the databases was limited, which may have led to inaccurate results. Additional evidence is needed at the protein level (e.g., from immunohistochemistry or Western blotting experiments) to verify that ATP6AP1 is differentially expressed between normal and cancerous breast tissues. Secondly, due to the limitations of the databases, we were not able to explore the relationship between *ATP6AP1* expression and immune infiltration in greater detail. Thirdly, the mechanism whereby *ATP6AP1* promotes a poor prognosis in BC was not determined. Future research is urgently needed to confirm our substantial results. In summary, *ATP6AP1* was significantly upregulated in BC tissues, and higher *ATP6AP1* expression was associated with a poorer prognosis and with higher or lower infiltration of particular immune cells in BC. The poorer prognoses of BC patients with higher *ATP6AP1* levels may have been due to the association of *ATP6AP1* with immune infiltration, and this possibility is worthy of further research. Tumor tissues may be especially prone to SARS-CoV-2 infections, which may downregulate *ATP6AP1*, ultimately impacting the prognoses of BC patients with COVID-19.

## MATERIALS AND METHODS

### Differential expression analysis

To compare *ATP6AP1* expression between BC and adjacent normal tissues, we used the Oncomine [37] (http://www.oncomine.org), GEPIA [38] (http://gepia.cancer-pku.cn/index.html) and HPA [39] (http://www.proteinatlas.org) databases. The Oncomine database draws relevant datasets directly from the Stanford Microarray Database, the National Center for Biotechnology Information Gene Expression Omnibus (GEO), published literature, etc. In Oncomine, mRNA data were selected with *P* = 0.05 and fold-change = 1.5 as the threshold values. The datasets in GEPIA are based on TCGA and GTEx, which contain normal tissue data for comparison.

The HPA database is a Swedish project to map all human proteins in cells, tissues and organs by integrating data from TCGA, HPA datasets, the GTEx consortium and recount2. We used the HPA database to assess ATP6AP1 protein levels in tumors and adjacent normal tissues. The antibody used to obtain the immunohistochemistry results was CAB015218 (Origene) at a dilution of 1:30. The immunohistochemistry results and antibody information are shown in Figure 1; however, due to the number of specimens in the database, we selected only a representative group for the figure, and placed the rest in the Supplementary Material.

GEO is a common functional genomics data repository. We analyzed expression profiles from GSE153277 [40] and GSE155241 [41]. GSE153277 contains nine samples from iAT2 cells infected with SARS-CoV-2 or a mock virus. GSE155241 contains data from hPSC-LO cells cultured with SARS-CoV-2 or a mock virus. Nine samples were selected from a total of 18. The GPL18573 Illumina NextSeq 500 (Homo sapiens) and GPL24676 Illumina NovaSeq 6000 (Homo sapiens) platforms were used to sequence the respective datasets. *ATP6AP1* levels were compared between the control and SARS-CoV-2 groups using the Sangerbox tool, a free online platform for data analysis (http://www.sangerbox.com/tool).

### Prognostic analysis

To assess the clinical significance of *ATP6AP1*, we used RNA sequencing data from Kaplan-Meier plotter [42] (https://kmplot.com) to evaluate the OS and RFS of BC patients based on *ATP6AP1* expression. Kaplan-Meier plotter includes data from GEO, the European Genome-Phenome Archive and TCGA. We also used data from GEPIA to determine the association of *ATP6AP1* expression with BC patient survival. In addition, we performed survival analyses based on *ATP6AP1* expression stratified by TIIC subtype levels in BC tissues.

### Immune infiltration analysis

The TIMER 2.0 database [14] (http://timer.cistrome.org/) is a comprehensive resource for the systematic analysis of immune infiltration in different cancer types based on data from TCGA. It integrates six state-of-the-art algorithms, including TIMER, xCell, MCP-counter, CIBERSORT, EPIC and quanTIseq. TISIDB [43] (http://cis.hku.hk/TISIDB/index.php) is a web portal for detecting tumor and immune system interactions. TISIDB integrates data from TCGA, PubMed literature, other public databases (UniProt, GO, DrugBank, etc.) and high-throughput sequencing analyses. The gene set variation analysis package was used to infer tumor-infiltrating lymphocyte levels in TISIDB. We further explored the relationship between *ATP6AP1* and TIIC levels in BC and analyzed the surface markers of TIICs using the CellMaker [44] (http://biocc.hrbmu.edu.cn/CellMarker/) database.

### Genomic analysis

To analyze the mutation and copy number variation of *ATP6AP1* in BC, we used the cBioPortal database [45, 46] (https://www.cbioportal.org), which contains numerous multidimensional cancer genomics datasets from various studies. The promoter methylation status of *ATP6AP1* and the associated clinical features were explored using UALCAN [47] (http://ualcan.path.uab.edu/index.html), a comprehensive, user-friendly, interactive web resource for analyzing cancer OMICS data (TCGA and MET500).

### Gene co-expression and functional enrichment analyses

We used the UALCAN database to determine the genes that were co-expressed with *ATP6AP1* in BC. The STRING database [48] (https://string-db.org) was used to construct the protein-protein interaction network of ATP6AP1 in BC. GO and KEGG enrichment analyses of ATP6AP1 and its interacting proteins were conducted using Metascape [49] (https://metascape.org), an integrated web-based portal.

### Network analysis

The lncRNAs, miRNAs and TFs associated with *ATP6AP1* were assessed using GCBI (https://www.gcbi.com.cn), a resource based on multiple databases and published studies.

### Statistical analysis

The log-rank test was used to analyze patients’ outcomes. The correlations of *ATP6AP1* levels with TIIC or immune cell marker levels were evaluated using Spearman's correlation analyses. The association of *ATP6AP1* expression with pathological staging was determined using one-way analysis of variance with data from GEPIA. Student’s *t*-test was used to analyze the association of *ATP6AP1* expression with clinical factors from UALCAN. Wilcoxon’s signed rank test was used to compare *ATP6AP1* levels between the control and SARS-CoV-2-infected groups. Spearman’s method was used to determine the correlation coefficients between genes in the co-expression analysis. All results with *P*-values <0.05 were considered statistically significant.

## Supplementary Materials

Supplementary Figures
