# Supplementary material for: Prognostic and immunological value of ATP6AP1 in breast cancer: implications for SARS-CoV-2
Source: Aging (Albany NY). 2021 Jul 6;13(13):16904–21. doi: 10.18632/aging.203229 (PMC8312471; doi:10.18632/aging.203229)
Supplement: Supplementary Figures [file aging-13-203229-s001.pdf]

SUPPLEMENTARY FIGURES

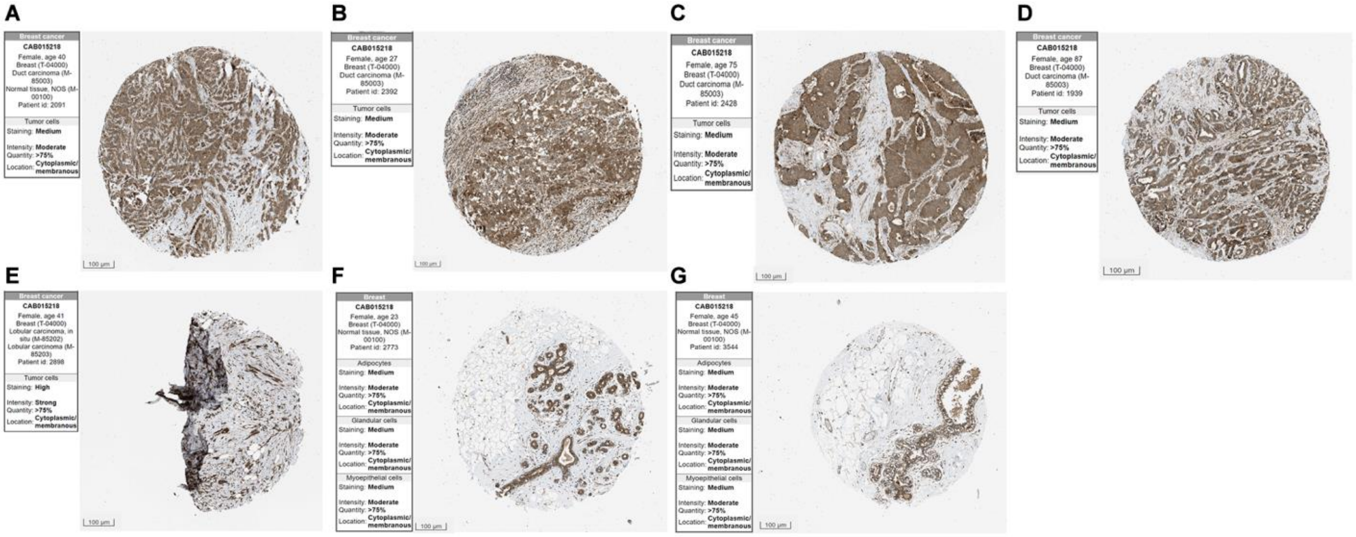

Supplementary Figure 1. ATP6AP1 protein levels in normal (**A**–**E**) and cancerous breast tissues (**F**, **G**) based on immunohistochemistry data from the HPA database (antibody: CAB015218, provided by Origene. Dilution: 1:30).

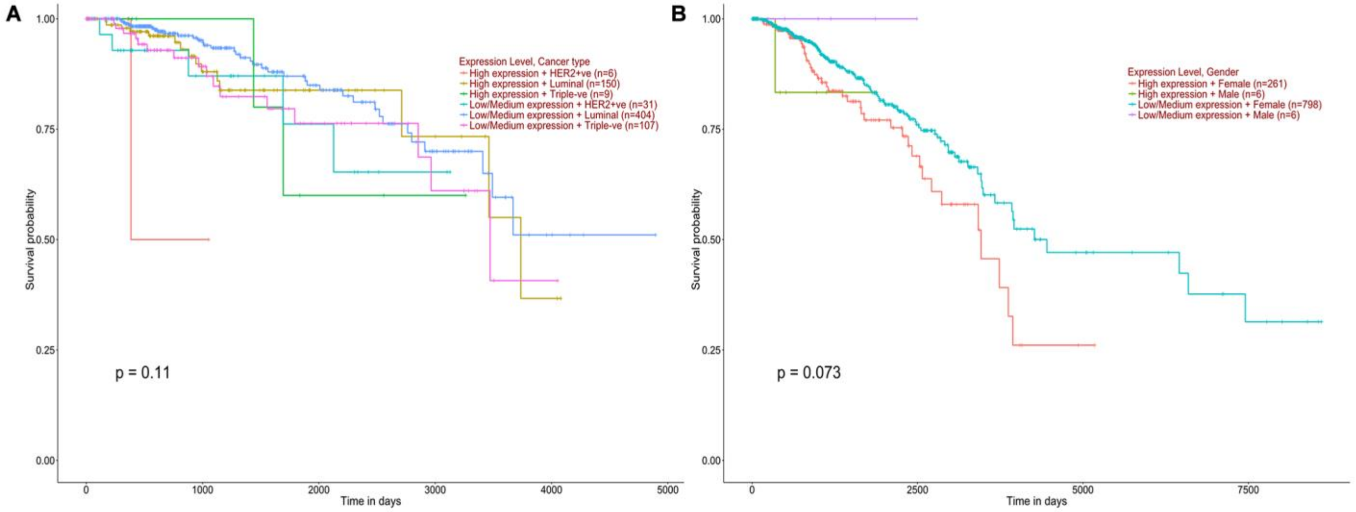

Supplementary Figure 2. The survival analysis of *ATP6AP1* expression based on subtype (**A**) and gender (**B**) of BC.

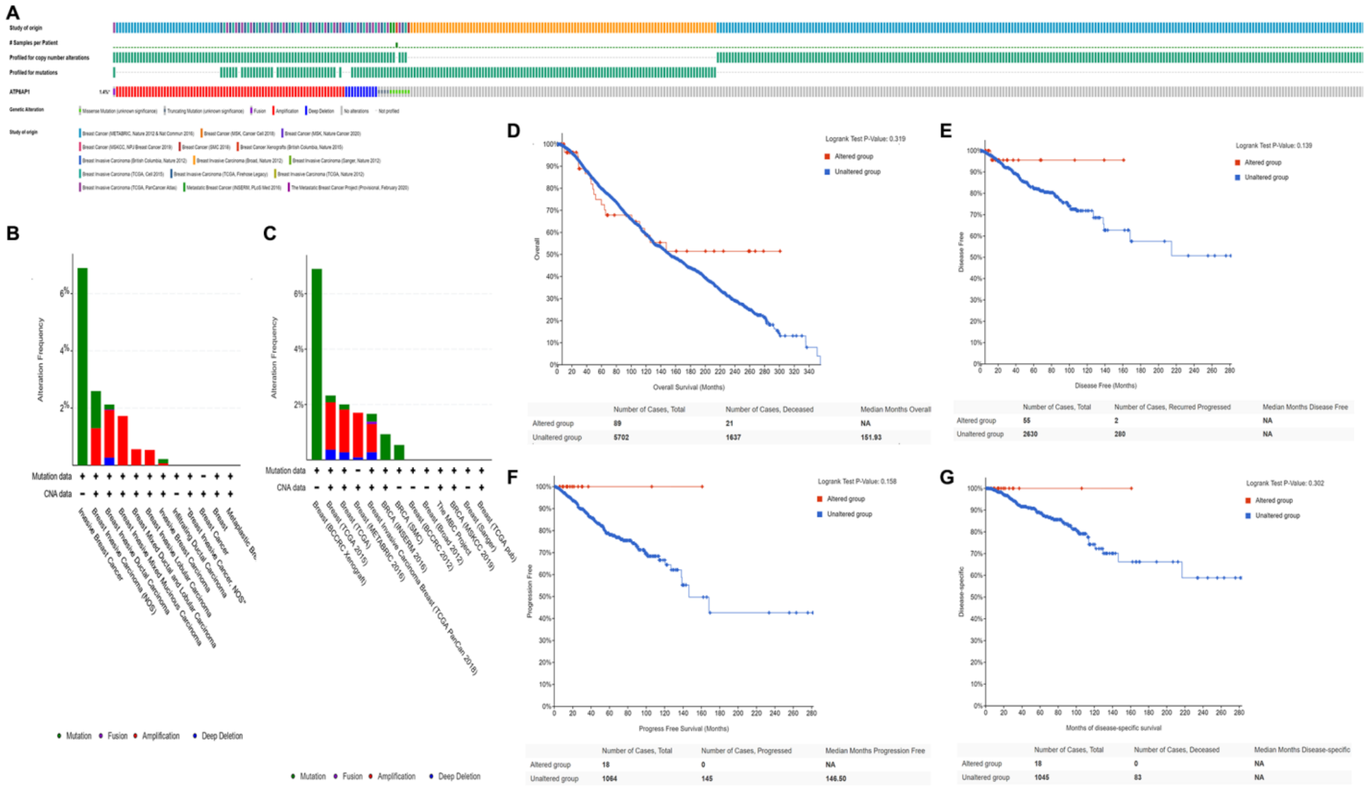

Supplementary Figure 3. The mutation analysis of ATP6AP1 in BC. (**A**) The genetic alternation condition of ATP6AP1 in BC (*n* = 7274). (**B**, **C**) The summary of alternation frequency based on different datasets and cancer types in BC (*n* = 7274). (**D**–**G**) The prognosis of ATP6AP1 based on its alternation condition.
